# Quantitative Limbic System Mapping of Main Cognitive Domains in Multiple Sclerosis

**DOI:** 10.3389/fneur.2018.00132

**Published:** 2018-03-12

**Authors:** Zafer Keser, Khader M. Hasan, Benson Mwangi, Kyan Younes, Mahsa Khayat-Khoei, Arash Kamali, John A. Lincoln, Flavia M. Nelson

**Affiliations:** ^1^Department of Neurology, McGovern Medical School, University of Texas Health Science Center at Houston, Houston, TX, United States; ^2^Department of Diagnostic and Interventional Radiology, McGovern Medical School, University of Texas Health Science Center at Houston, Houston, TX, United States; ^3^Department of Psychiatry, McGovern Medical School, University of Texas Health Science Center at Houston, Houston, TX, United States

**Keywords:** limbic system, cognition, multiple sclerosis, diffusion tensor imaging, cortical thickness

## Abstract

**Background and objective:**

Cognitive impairment (CI) is common in multiple sclerosis (MS), but underlying mechanisms and their imaging correlates are not completely understood. The gray and white matter structures of the limbic system (LS) play crucial roles in different aspects of cognition. To investigate their role in MS related CI, and since a detailed evaluations are lacking in the literature, we used a comprehensive neuroimaging approach to evaluate CI’s correlations with the main components of the LS.

**Methods:**

Ten non-cognitively impaired MS patients and 30 MS patients with diagnosed CI, who underwent a comprehensive neuropsychological evaluation were included in the analysis. Microstructural integrity, volumetry of main limbic gray and white matter structures and cortical thickness were assessed for associations with CI.

**Results:**

Fornix and cingulum/cingulate cortices were found to be the strongest correlates of CI in MS. As expected, LS’ gray and white matter structures were involved in various cognitive functions. Uncinate fasciculi showed significant correlation with verbal and visuospatial learning and memory, phonemic and semantic fluency; hippocampi with visuospatial skills, phonemic and semantic fluency, executive functions, and processing speed; thalami with verbal learning, visuospatial skills, semantic fluency; and amygdala with verbal recognition discrimination.

**Conclusion:**

This comprehensive neuroimaging approach elucidated the role of the main limbic structures in cognitive functions associated with MS-related CI.

## Introduction

Cognitive impairment (CI) occurs in 40–65% of patients with multiple sclerosis (MS) (1), mainly encompassing disturbances in memory, attention, verbal fluency (VF), information processing speed, conceptual reasoning, and visuospatial perception (2). Damage to white matter structures from demyelination or axonal loss leading to disconnection between the cortical and subcortical regions responsible for cognition, underlies the cognitive symptomatology in MS (3, 4). Although MS was originally thought to be mainly a white matter disease, gray matter structures are also known to be involved in the disease pathogenesis and shown to correlate with impairment (5, 6).

**Figure 1:**
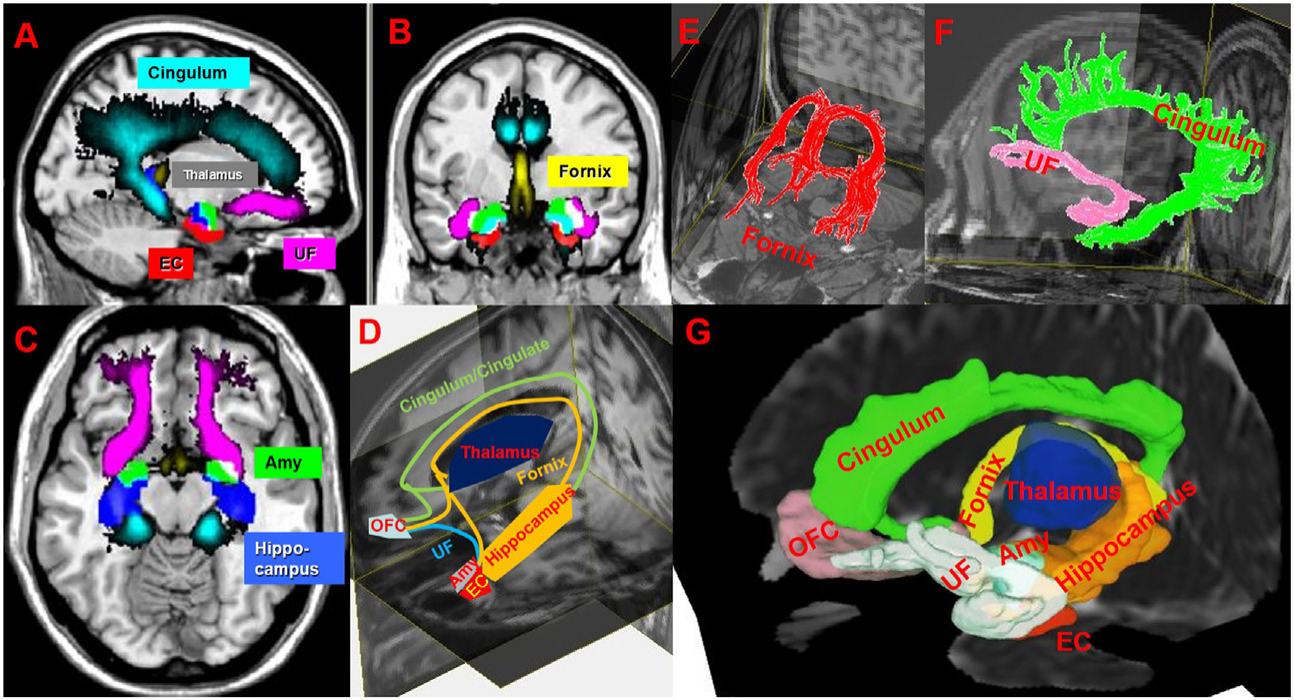
**(A–C)** Illustration of spatial-normalized MNI summary of cingulum, fornix, uncinated fasciculus (UF), thalamus, entorhinal cortex (EC), amygdala (Amy) in sagittal, coronal and axial T1w slices in our multiple sclerosis (MS) cohort. **(D,G)** Schematic **(D)** and atlas-based **(G)** representation (in one MS patient) of limbic connections between limbic gray matters on 3D T1w and mean diffusivity images. **(E,F)** Illustrates cingulum (green), UF (pink), and fornix (red) on T1w. Abbreviation: OFC, orbitofrontal cortex. MRIcron (http://people.cas.sc.edu/rorden/mricron/index.html), Diffusion tensor imaging (http://cmrm.med.jhmi.edu/), and DSI (http://dsi-studio.labsolver.org/) studios are used to generate this figure.

The limbic system (LS) includes gray matter and white matter structures that along with paralimbic structures, play a crucial role in different aspects of cognition (7). Hippocampus, thalamus, and the ventral cingulum connected by the fornix, are responsible for memory and spatial orientation, whereas the amygdala, orbitofrontal cortex (OFC), entorhinal cortex (EC), and parahippocampal (PH) cortex connected by uncinate fasciculus, are involved in multimodal sensory integration, behavioral inhibition, the reward-pleasure system, and memory for visual information (8). On the other hand, the cingulate gyrus and cingulum are associated with attention, response selection/action monitoring, self-knowledge, and reasoning (9) (see Figure 1).

The LS recently received more attention in the pursuit of understanding the neural correlates of abnormal cognitive symptomatology in MS (4, 10). The LS’s white (11, 12) and gray matter structures (13, 14) are found to be implicated in various cognitive symptoms in MS. Of note, compared with healthy controls, reduced cerebral blood flow was present in limbic regions of patients with CI (15). Although most studies focus on individual parts of LS, a comprehensive evaluation that included all components of the LS is lacking (see Table 1 for a summary of key MRI studies investigating limbic structures in MS).

**Table 1:** Tabulated summary of key MRI studies investigating limbic structures in multiple sclerosis (MS).

Author name	Structures	Clinical test	MRI technique	Number of subjects	Results/notes
Batista et al. (14)	Whole gray matter structures	PASAT, SDMT, CVLT, COWAT, BVMT, D-KEFS	T1w-volumetric segmentation	59 RRMS 27 SPMS	Thalamus-SDMT was found to be correlate with CI, correlations were adjusted for age and neocortical volume
Benedict et al. (45)	All GM structures by Freesurfer	SDMT, CVTL-II, BVMT	T1w-Freesurfer	35 RRMS 15 SPMS	Thalamus showed some correlation with almost all cognitive scores but strong correlation was noted between Thalamus-SDMT, amydala-recognition DI
Dineen et al. (3)	Cingulum, Fornix, UF	MACFIMS	DTI-TBSS	35 RRMS 2 SPMS	Associations between left cingulum FA and PASAT, left cingulum FA, and fornix FA and BVRT, and left cingulum FA and fornix FA CVLT-II
Fink et al. (50)	Fornix, UF, cingulum	CVLT	DTI-deterministic tractography	50 RRMS	Right fornix RD were associated with delayed recognition
Hojjat et al. (52)	Whole brain	MACFIMS	pCASL	39 RRMS	Decreased blood flow in bilateral cingulate gyri in cognitively impaired MS
Houtchens et al. (13)	Thalamus	COWAT, JLO, CVLT-II, PASAT, BVMT, SDMT	T1w/T2w-manual delineation	62 RRMS, 16 SPMS, 1 PPMS	Thalamus volume was strongly correlated with COWAT, JLO, CVLT-II, BVMT, PASAT, SDMT
Kern et al. (12)	Fornix, UF, cingulum, thalamus, and hippocampus	WAIS III, the D-KEFS, SDMT, PASAT the BSRT, and the spatial-recall task	DTI-deterministic tractography	27 RRMS	Group instead of correlation analyses were used. Attention, verbal memory, closely associated with thalamic volume and processing speed, spatial memory associated with UF FA
Meijer et al. (10)	Cingulum, UF, fornix	PASAT-3, SDMT SRT, RMT, FRT, WAIS III, Stroop color-word interference test, and Hayling Sentence Completion Test	TBSS-DTI	32 SPMS	Limbic pathways were associated with visuospatial memory
Mesaros et al. (43)	UF and cingulum	PASAT SDMT SPART-D SPART-T SRT-C SRT-D, SRT-L, WLG	DTI-Atlas-based	40 RRMS 19 SPMS 23 PPMS	Cingulum DTI metrics were the best classifiers across numerous tests: PASAT, SDMT, SRT, and WLG
Pardini et al. (47)	FSL segmentation of hippocampus	Brief repeatable neuropsychological battery	T1w-FSL	25 RRMS	Verbal memory correlated with left hippocampal volume, spatial memory with right hippocampal volume, memory deficits with left cingulum, and left UF’ mean FA
Pravatà et al. (48)	All GM structures by Freesurfer	Brief repeatable battery of neuropsychological tests	T1w-FreeSurfer	108 RRMS, 14 SPMS, 4 PPMS	Bilateral entorhinal, right OFC, right cingulate and bilateral, temporal poles were associated with CI. (correlations not adjusted for age, lesion load, and education)
Riccitelli et al. (44)	Whole brain thru VBM	PASAT 3, short story, verbal learning, recall-ROCF, verbal fluency, spatial cognition-ROCF	T1/T2w-VBM	22 RRMS, 29 SPMS, 22 PPMS	MS with CI vs HC had GM reduction in the hippocampi, right insula, cingulate cortex. GM loss in the left hippocampus was correlated with CI index. Anterior cingulate cortex was found to separate MS with cognitively intact MS vs. CI

*BSRT, Buschke Selective Reminding Test; BVMT-R, Brief Visuospatial Memory Test-Revised; BVRT, Benton Visual Retention Test; CI, cognitive impairment; COWAT, the controlled oral word association test; CVLT-II, California Verbal Learning Test II; D-KEFS, the Delis–Kaplan Executive Function System; DTI, Diffusion Tensor Imaging; FA, fractional anisotropy; FRT, Free recall test; GM, gray matter; JLO, Judgment of Line Orientation test; MACFIMS, minimal assessment of cognitive function in MS; OFC, orbitofrontal cortex; PASAT, Paced Auditory Serial Addition Test; pCASL, pseudo-continuous arterial spin labeling; PPMS, primary progressive MS; RD, radial diffusivity; RMT, Recognition Memory Test; ROCF, The Rey–Osterrieth complex figure test; RRMS, relapsing–remitting MS, SDMT, Symbol Digit Modality Test; SPART-D, Spatial Recall Test–delayed recall; SPART-T, Spatial Recall Test–immediate recall; SPMS, secondary–progressive MS, SRT-C, Selective Reminding Test; SRT-D, Selective Reminding Test–delayed recall; SRT-L, Selective Reminding Test–long-term storage; T1w, T1 weighted; T2w, T2 weighted; TBSS, tract-based spatial statistics; UF, uncinate fasciculus; VBM, voxel-based analysis; WAIS-III, Wechsler Adult Intelligence Scale-III; WLG, Word list generation*.

Several studies have shown correlation between various MRI measure and histopathology (16, 17, 18). Diffusion tensor imaging (DTI) metrics of white matter tracts were found to correlate with a histologically derived measure of tract myelination (17, 18). It is suggested that fractional anisotropy (FA) and mean diffusivity (MD) of white matter tracts are affected by axonal count in *post mortem* analyses in MS (18). Additionally, cortical thickness measures derived from MRI and histological measurements showed significant correlations in MS (17). While no specific postmortem DTI studies have been detailed for limbic structure damage in MS, cortical thickness (19) and diffusion measurements are a reliable *in vivo* technique to quantify gray and white matter injury in MS (18).

In this study, we attempted to provide a comprehensive analysis of the roles of the LS’s gray and white matter structures on the main cognitive functions affected by MS. We used quantitative MRI (qMRI) methods such as cortical thickness and volumetric analysis of deep and cortical gray matter structures derived from T1-weighted (T1w) images. In addition, we used microstructural measures such as FA and MD derived from DTI. Cognitive scores were obtained from subsets of the Minimal Assessment of Cognitive Function in MS (MACFIMS), a comprehensive cognitive battery assessing various cognitive domains specifically related to MS (20).

## Materials and Methods

Ten non-cognitively impaired MS patients (MSNI) and 30 patients with diagnosed CI (MSCI) were included (36 relapsing–remitting, and 4 secondary-progressive) age 40.53 ± 11.53 (range 18–58) years, education 14.65 ± 2.33 years, disease duration 13.71 ± 8.82 years, Expanded disability status scale 3.21 ± 1.89 (0–7), left handedness 3/40 (Table 2). Written informed consent was obtained from each subject following University of Texas Health Science Center Institutional Review Board approval of the protocol. All patients underwent the comprehensive cognitive testing as detailed below. Cognitive testing was performed in the morning to avoid fatigue, prior to and within 2 weeks of the imaging session. Inclusion criteria specified meeting 2010 McDonald Criteria for MS. Exclusion criteria included history of psychiatric disorders, recent history of drug or alcohol abuse, history of depression or relapse within 3 months of enrollment, history of allergy to gadolinium, history of other brain pathology, claustrophobia, or positive urine pregnancy test prior to MRI.

**Table 2:** Subject characteristics, MRI derived values of the brain structures, and cognitive scores investigated in this study.

	Mean	SD
Age	40.53	11.53
Expanded Disability Status Scale	3.21	1.89
Disease DURATION	13.71	8.82
Education	14.65	2.33
T1 black holes (mL)	3.95	3.65
T2 hyperintensities (mL)	14.48	13.15
Fornix volume	3.23	1.35
Fornix FA	0.33	0.03
Fornix MD	1.38	0.11
Left cingulum volume	11.10	2.44
Left cingulum FA	0.36	0.02
Left cingulum MD	0.81	0.04
Right cingulum volume	10.45	3.03
Right cingulum FA	0.34	0.02
Right cingulum MD	0.80	0.05
Left uncinate volume	4.97	2.74
Left uncinate FA	0.37	0.02
Left uncinate MD	0.85	0.07
Right uncinate volume	4.90	2.05
Right uncinate FA	0.35	0.02
Right uncinate MD	0.84	0.05
Left thalamus volume	7.09	1.40
Left thalamus MD	0.88	0.05
Right thalamus volume	6.38	1.29
Right thalamus MD	0.88	0.04
Left hippocampus volume	3.66	0.58
Left hippocampus MD	0.95	0.02
Right hippocampus volume	3.77	0.52
Right hippocampus MD	0.94	0.03
Left amygdala volume	1.42	0.29
Left amygdala MD	0.88	0.05
Right amygdala volume	1.53	0.27
Right amygdala MD	0.88	0.04
LH lateralorbitofrontal thickness	2.45	0.17
LH medialorbitofrontal thickness	2.29	0.15
RH lateralorbitofrontal thickness	2.46	0.18
RH medialorbitofrontal thickness	2.32	0.15
LH caudalanteriorcingulate thickness	2.44	0.25
LH isthmuscingulate thickness	2.27	0.20
LH posteriorcingulate thickness	2.37	0.18
LH rostralanteriorcingulate thickness	2.76	0.22
RH caudalanteriorcingulate thickness	2.45	0.21
RH isthmuscingulate thickness	2.22	0.20
RH posteriorcingulate thickness	2.34	0.14
RH rostralanteriorcingulate thickness	2.81	0.25
LH parahippocampal thickness	2.56	0.27
RH parahippocampal thickness	2.52	0.24
LH entorhinal thickness	3.02	0.41
RH entorhinal thickness	3.18	0.43
CVLT_Total Correct	45.88	12.76
CVLT_CVLT_List B	5.12	2.32
CVLT_SDFR Score	8.43	4.12
CVLT_SDCR Score	10.02	3.77
CVLT_LDFR Score	8.88	4.31
CVLT_LDCR Score	10.16	3.69
CVLT_Hits	13.73	2.48
CVLT_False Positives	4.08	3.18
CVLT_Repetitions	3.73	3.27
CVLT_Intrusions	6.32	6.19
SDMT_Written	40.02	12.53
SDMT_Oral	46.41	14.85
PASAT_3	37.41	14.32
PASAT_2	28.43	12.65
BVMT_TotalRecallRaw	18.92	8.83
BVMT_LearningRaw	3.57	2.23
BVMT_DelayedRaw	7.31	3.64
BVMT_Percent_RetainedRaw	86.12	24.99
BVMT_RecognitionHitsRaw	5.10	1.01
BVMT_RecognitionFalseAlarmsRaw	0.53	0.44
BVMT_Recognition_DiscriminationIndexRaw	4.57	1.41
BVMT_RecognitionResponseBiasRaw	0.44	0.20
COWAT_TotalFAS_Raw	33.10	13.49
COWAT_Animals_Raw	15.71	5.24
JLO_FormH_Raw	19.98	6.50
JLO_FormH_Freq	16.14	11.88
JLO_FormV_raw	19.41	6.31
JLO_Form_V_freq	16.16	11.81
WCST_NumberCategories	5.14	1.61
WCST_TrialsAdministered	100.35	21.87
WCST_TotalCorrect	71.82	10.84
WCST_Trialsto1st	22.27	21.79
WCST_PerseverativeErrors	12.96	10.80
WCST_NonPerseverativeErrors_Raw	15.57	12.05
WCST_FailureToMaintainSet	0.88	0.77
CVLT_TotalCorrect_t	44.51	14.39
CVLT_ListB_zscore	−0.64	1.12
CVLT_SFDR_zscore	−0.96	1.49
CVLT_SCDRcore_zscore	−0.97	1.58
CVLT_LDFR_zscore	−1.09	1.68
CVLT_LDCR_zscore	−0.94	1.49
CVLT_Hits_zscore	−1.04	1.57
CVLT_FalsePositives_zscore	−0.80	1.78
SDMT_Written_zscore	−1.41	1.39
SDMT_Oral_zscore	−1.31	1.44
PASAT_3_zscore	−1.22	1.48
PASAT_2_zscore	−0.90	1.25
BVMT_TotalRecall_t	38.37	14.92
BVMT_Learning_t	49.41	12.75
BVMT_Delayed_t	40.22	15.75
VF_TotalFAS_t	39.92	11.80
VF_Animals_t	35.51	11.73
Benton_FormH_Percentile	35.96	32.20
Benton_Form_V_percentile	32.41	28.81
WCST_NumberCategories_percentile	13.33	4.81
WCST_Trialsto1st_percentile	10.63	6.24
WCST_PerseverativeErrors_t	47.80	11.61
WCST_NonPerseverativeErrors_t	43.45	9.63

*Volumes [mean and SD are expressed in milliliter (mL)], thicknesses in millimeter (mm), fractional anisotropy (FA) in μ ± σ, and mean diffusivities (MD) in ×10^−3^ mm^2^ s^−1^ for right (RH) and left (LH) hemispheres. 2s, 2 s tests; 3s, 3 s, BVMT, Brief Visuospatial Memory Tests; COWAT, Controlled Oral Word Association Test; CVLT, California Verbal Learning Test 2nd version; JLO, Benton Judgment Line of Orientation; LDCR, long-delayed cue recall; LDFR, long delayed free recall; PASAT, Paced Auditory Serial Addition Test; SDCR, short-delayed cue recall; SDFR, Short-Delayed Free Recall; SDMT, symbol digit modality test; t, t test; z, z scores; WCST, Wisconsin Card Sorting Test*.

### Behavioral Laboratory Measures

In order to determine CI in our cohort, an impairment index methodology was applied to the behavioral scores. Based on previously validated methodology (20), 20 MACFIMS parameters were identified as most pertinent in the measurement of MS-related cognitive deficits and a CI index was derived. The processing speed and working memory was evaluated by using Paced Auditory Serial Addition Test (PASAT) (21) and symbol digit modality test (SDMT) (22), memory and learning evaluated by using California Verbal Learning Test Second Edition (CVLT-II) (23, 24) and Brief Visuospatial Memory Test-Revised (25), executive function using Wisconsin Card Sorting (26), visual perception/spatial processing using Judgment of Line Orientation test (JLO) (27), and VF measured by the controlled oral word association test (COWAT) (27). Patients were classified as cognitive impaired if their performance was more than one standard deviation below the mean on at least 40% of the pre-identified parameters and classified as cognitively intact if performance was less than 40% impaired.

### Magnetic Resonance Imaging Data Acquisition

Whole brain MRI data were acquired on a Philips 3.0T Intera scanner using a SENSE receive head coil. The MRI protocol included conventional and non-conventional MRI sequences [dual echo turbo spin echo, fluid attenuation by inversion recovery (FLAIR) and 3D T1-weighted magnetization prepared rapid acquisition with gradient echo (MPRAGE)]. The T1-weighted sequence spatial resolution was 1 mm × 1 mm × 1 mm and field-of-view was 256 mm × 256 mm. Diffusion-weighted image (DWI) data were acquired axially from the same graphically prescribed conventional MRI volumes using a single-shot multi-slice 2-D spin-echo diffusion sensitized and fat-suppressed echo planar imaging (EPI) sequence, with the balanced Icosa21 tensor encoding scheme (28, 29). The *b*-factor = 1,000 s mm^−2^, TR/TE = 7,100/65 ms, FOV = 256 mm × 256 mm, and slice thickness/gap/#slices = 3 mm/0 mm/44. The EPI phase encoding used a SENSE k-space undersampling factor of two, with an effective k-space matrix of 128 × 128, and an image matrix after zero-filling of 256 × 256. The constructed image spatial resolution for the DWI data was = 1 mm × 1 mm × 3 mm.

### Lesion Load (LL) Segmentation using Conventional MRI

Whole brain LL was quantified in all patients using the co-registered multispectral dual FSE and the FLAIR volumes. The lesion probability masks were computed in MRIcron (http://www.nitrc.org/projects/mricron/) (30, 31). The lesion volumes were saved as binary masks to enable fusion with other multimodal volumes acquired from the same subject. We obtained both T1 and T2 LL to adjust for in the correlation analyses.

### Diffusion Tensor White Matter Tractography

**Figure 2:**
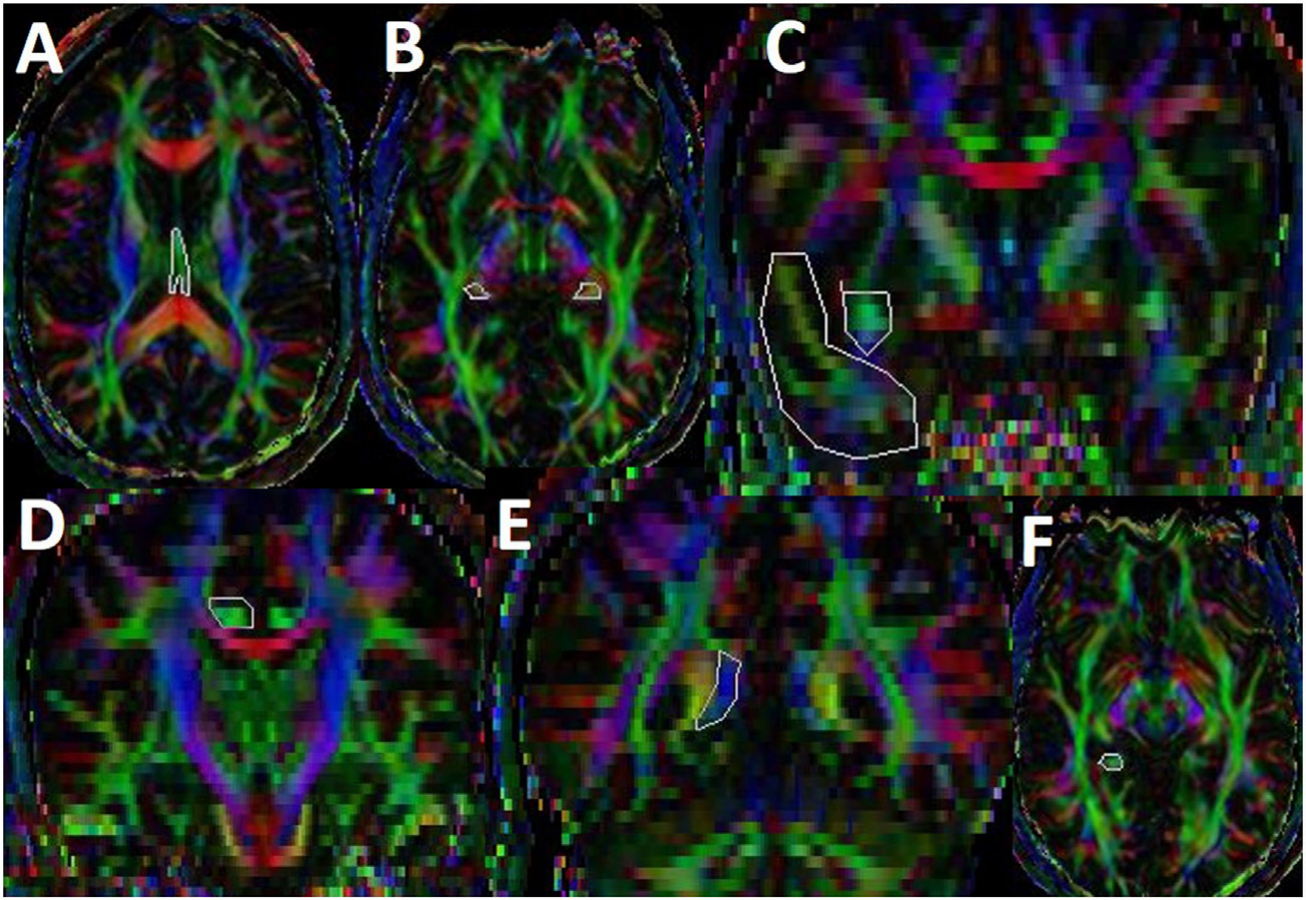
Illustration of fiber tractography in color-coded diffusion tensor imaging map. For fornix, we have seeded first ROI in subcallosal green fibers seen in axial slice where body and crus of fornix can be observed **(A)** and second and third ROIs are seeded in the mid and distal parts of crus (B) (35). For uncinate fasciculus (UF), we have seeded first ROI in the coronal section of temporal lobe and second ROI in the superomedial green projections fibers at the anterior commissure level **(C)** (34). For cingulum, we have seeded first ROI and second ROI in supracallosal green projection fibers anteriorly **(D)** and posteriorly **(E)**. Third ROI was seeded in the green hippocampal cingulum fibers seen in the medial temporal lobe **(F)** (4). Once a fiber tract was reconstructed, its entire trajectory was verified on a slice-by-slice basis to compare with established anatomical landmarks described in the human brain neuroanatomy atlases (33).

We used a brute force and multiple regions-of-interest (ROI) tracking method and the fiber assignment with continuous tractography (FACT) algorithm (32, 33) (DTI Studio, Johns Hopkins University, Baltimore, MD, USA) to reconstruct fornix, cingulum, and uncinated fasciculus with a FA threshold of 0.15 and an angle threshold of 70°. Reproducibility of the fiber construction in both hemispheres was tested on all subjects by two experienced raters (Zafer Keser and Khader M. Hasan). We used color-coded principal eigenvector red–green–blue (RGB) map derived from DTI to seed ROIs. Multiple ROI-based deterministic tractography was used for fornix, cingulum, and uncinate fasciculus as described in Figure 2 (4, 34–37) (Figure 2).

### Tissue Segmentation and Parcelation Using T1-Weighted and DTI Data

Using FreeSurfer software library (version 5.3) (38), the T1-weighted brain data were automatically segmented into cerebellar, brainstem, brain, and cerebrospinal fluid, which also included thalamus, amygdale, and hippocampus (39). DTI-derived data volumes (FA, mean, axial, and radial diffusivities) were coregistered to the T1-weighted volume to obtain diffusivity values for gray matter limbic structures. In brief, all the T1-weighted data were visually inspected to rule out artifacts and input to FreeSurfer’s “recon-all” routine for segmentation and extraction of morphometric measurements. FreeSurfer provided average cortical thickness using the cortical atlas labels described elsewhere (40).

### Statistical Analyses

We used raw scores for cognitive scores. We tested normality using the Kolmogorov–Smirnov test for all reported measures. As our data couldn’t assure normal distribution for all the variables likely due to small sample size, we used non-parametric Spearman correlation analyses. For the whole cohort (*n* = 40), individual behavioral scores from each component of the MACFIMS were adjusted for age, years of education, and total brain lesion volume (T1 hypointensities + T2 hyper intensities without double counting), but not for handedness. They were then, computed with all qMRI measures using the partial Spearman rank correlations. We used normalized volumetric measures scaled for each subject by the individual estimated intracranial volume. All analyses and generation of scatter plots were performed in SPSS Statistics 24.0-IBM software. Significance defined if *p* < 0.05. We used Bonferroni corrections for multiple comparisons. After multiple comparisons, significance defined as *p* < 0.00003.

## Results

**Figure 3:**
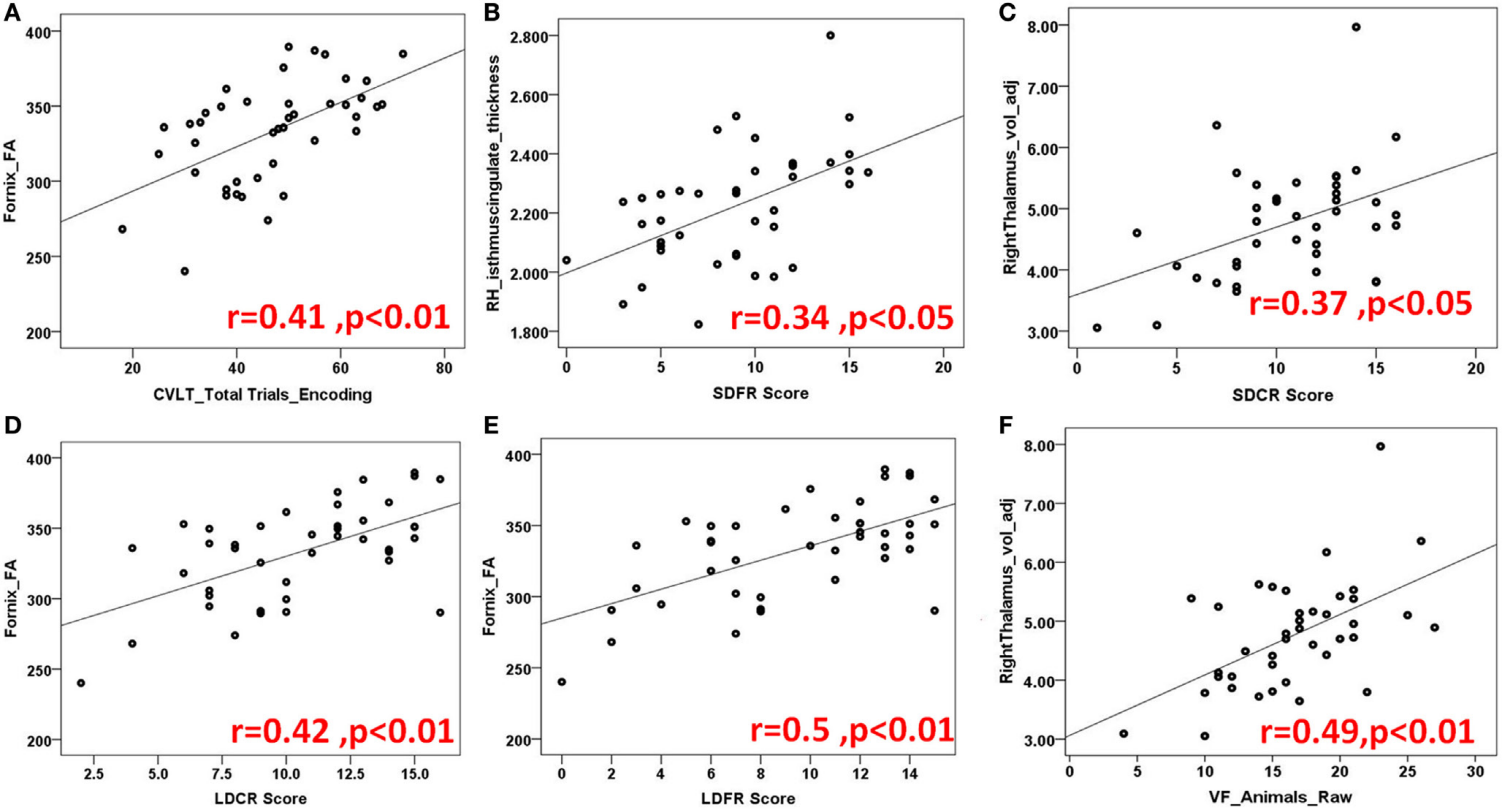
Demonstration of several scatter plots of limbic structures and California verbal learning (CVLT) **(A–E)** and controlled word association test [semantic verbal fluency (VF)] **(F)**. Abbreviations: FA, fractional anisotropy; RH, right hemisphere; SDFR, Short-Delayed Free Recall; SDCR, short-delayed cue recall; LDCR, Long-delayed cue recall; LDFR, Long Delayed Free Recall; voladj, volume adjusted for intracranial volume.

**Figure 4:**
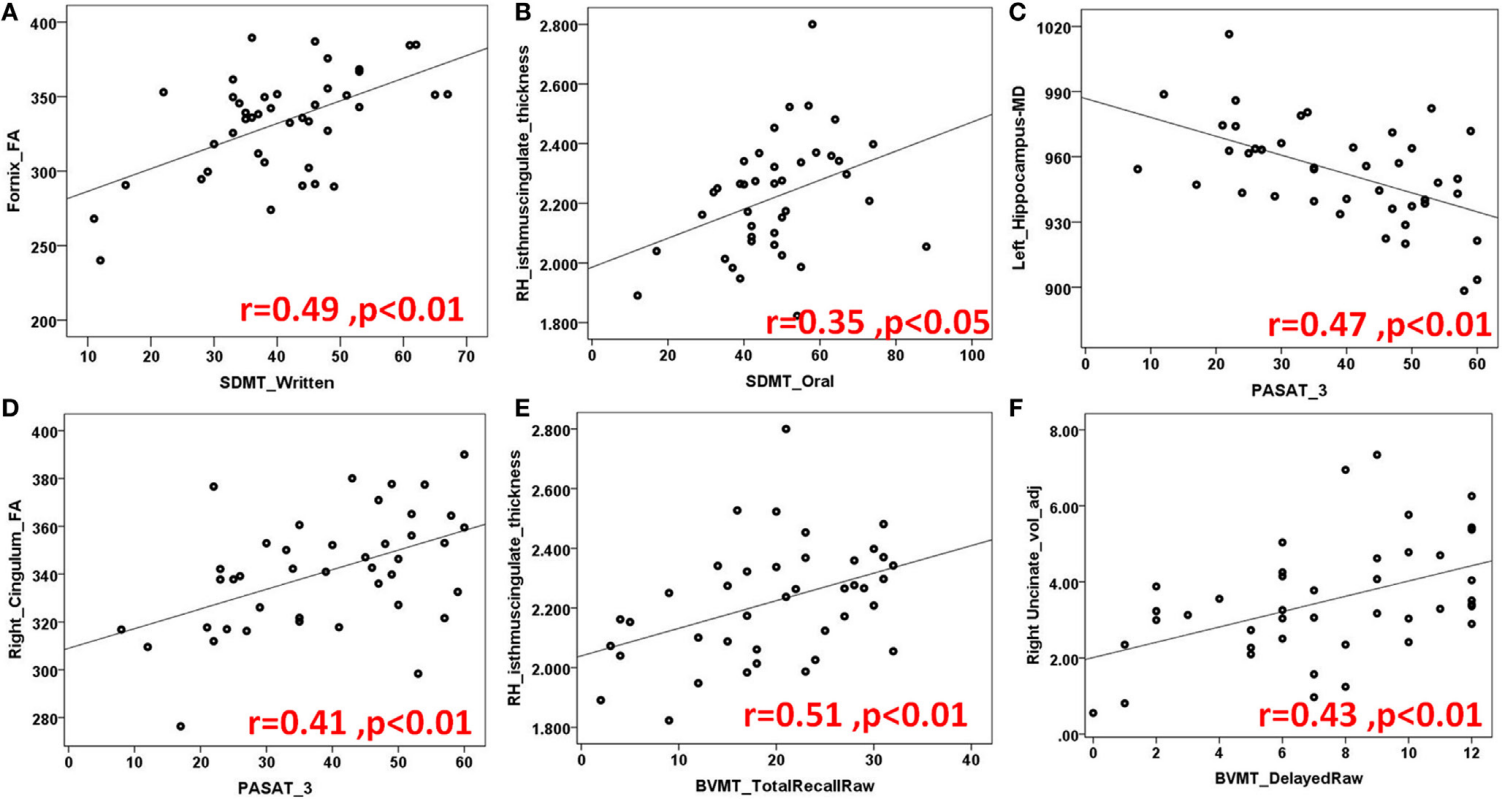
Another demonstration of several scatter plots of limbic structures and Symbol Digit Modality Test (SDMT) **(A,B)** written and oral versions, Paced auditory serial addition test (PASAT) **(C,D)**, Brief visuospatial memory test (BVMT) total **(E)** and delayed recall **(F)** sets. Abbreviations: FA, fractional anisotropy; MD, mean diffusivity; RH, right hemisphere; voladj, volume adjusted for intracranial volume.

Figures 3 and 4 highlight scattered plots with the best fit line curves for the some of the most notable correlations. Table 3 shows all the significant correlations with *r* and *p*-values. Most of the significant correlations did not survive multiple comparisons except the correlations of fornix FA with long delayed free recall in CVLT-II, SDMT oral test, and COWAT animals; left cingulum FA-PASAT 2 s, left hemisphere rostral anterior cingulate-form H in JLO and left hippocampus MD-form H in JLO.

**Table 3:** Significant correlations are shown for California Verbal Learning Test 2nd version (CVLT_II), Brief Visuospatial Memory Tests (BVMT), symbol digit modality test (SDMT) and Paced Auditory Serial Addition Test (PASAT) 3 seconds (3 s) and 2 second (2 s) tests, Controlled Oral Word Association Test (COWAT), Benton Judgment Line of Orientation (JLO), Wisconsin Card Sorting Test (WCST).

CVLT_II				BVMT		COWAT	
Trials 1–5	*r*-Values	Hits	*r*-Values	Total recall	*r*-Values	FAS	*r*-Values
For_FA	0.406**	R_Cing_MD	0.369*	L_Cing_MD	−0.318*	For_FA	0.377**
L_UF_FA	0.298*	R_UF_vol	−0.291*	R_UF_FA	0.332*	L_Cing_FA	0.325*
R_UF_vol	0.309*	L_Tha_vol	−0.349*	L_PH_th	−0.319*	L_UF_MD	−0.338*
R_iCC_th	0.370*	R_Amy_vol	−0.328*	R_caCC_th	0.333*	R_iCC_th	0.327*
L_PH_th	−0.378*	False Positives		For_FA	0.326*	L_HippMD	−0.382*
R_UF_vol	0.393**	For_FA	−0.412**	R_iCC_th	0.511**	L_caCC_th	0.460**
R_iCC_th	0.455**	L_Tha_vol	−0.371*	Learning		L_raCC_th	0.551**
R_Tha_vol	0.496**	R_Tha_vol	−0.352*	R_Cing	−0.303*	R_iCC_th	0.389*
SFDR		L_caCC_th	−0.326*	L_UF_FA	−0.325*	L_Cing_MD	−0.368*
For_FA	0.379**	L_raCC_th	−0.335*	Delayed Recall		R_Cing_FA	0.421**
R_Tha_vol	0.349*	R_iCC_th	−0.366*	R_UF_vol	0.431**	R_Cing_MD	−0.350*
R_caCC_th	0.338*	R_ThaMD	0.336*	R_caCC_th	0.371*	L_UF_FA	0.288*
R_iCC_th	0.421**	Repetitions		R_iCC_th	0.374*	L_UF_MD	−0.315*
SDCR		L_pCC_th	−0.360*	Retained Percent		R_UF_vol	0.407**
For_FA	0.436**	Intrusions		For_MD	0.395**	R_UF_FA	0.315*
R_Tha_vol	0.368*	R_UF_FA	−0.308*	L_Cing_vol	0.407**	R_UF_MD	−0.435**
R_iCC_th	0.388*	R_Tha_vol	−0.318*	False Alarms		R_Tha_vol	0.326*
LDCR		R_Amy_vol	−0.327*	R_mOFC_th	−0.387*	L_Hipp_vol	0.355*
For_FA	0.419**	R_lOFC_th	−0.325*	L_iCC_th	−0.399*	R_Amy_vol	0.331*
R_iCC_th	0.405**	R_mOFC_th	−0.340*	L_pCC_th	−0.368*	R_iCC_th	0.387*
LDFR		L_iCC_th	−0.358*	L_raCC_th	−0.384*	Animals	
For_FA	0.500**	L_raCC_th	−0.388*	R_caCC_th	−0.380*	For_FA	0.562**
L_Tha_vol	0.356*	R_caCC_th	−0.458**	R_iCC_th	−0.487**	R_Cing	0.380**
R_Tha_vol	0.381*	R_iCC_th	−0.505**	R_pCC_th	−0.506**	R_UF_MD	−0.339*
L_caCC_th	0.337*	R_pCC_th	−0.338*	R_PH_th	−0.317*	L_Tha_vol	0.414**
L_raCC_th	0.323*	R_PH_th	−0.377*	DI		R_Tha_vol	0.488**
R_caCC_th	0.323*	L_EC_th	−0.380*	R_iCC_th	0.325*	R_caCC_th	0.344*
R_iCC_th	0.437**	L_ThaMD	0.367*			R_iCC_th	0.426**
		R_ThaMD	0.315*			L_HippMD	−0.389*
		L_AmyMD	0.367*				

**JLO**		**PASAT**		**WCST**		**SDMT**	
**H Form**	*****r***-Values**	**3 s**	*****r***-Values**	**Number of categories**	*****r***-Values**	**Written**	*****r***-Values**

For_FA	0.458**	For_FA	0.366*	R_Cing_FA	−0.379**	For_FA	0.484**
R_UF_vol	0.297*	L_Cing_FA	0.369*	L_Hipp_vol	0.413**	L_lOFC_th	0.326*
R_UF_MD	−0.350*	R_Cing_FA	0.412**	Correct		R_iCC_th	0.333*
R_Tha_vol	0.346*	R_Cing_MD	−0.415**	L_Cing_MD	0.348*	Oral	
L_lOFC_th	0.336*	L_UF_FA	0.389**	L_caCC_th	0.364*	For_FA	0.502**
L_caCC_th	0.489**	L_UF_MD	−0.298*	R_pCC_th	0.349*	R_Cing_FA	0.313*
L_iCC_th	0.329*	R_UF_FA	0.302*	Trial to1st		L_lOFC_th	0.341*
L_raCC_th	0.544**	R_UF_MD	−0.366*	For_FA	−0.371*	R_iCC_th	0.353*
R_caCC_th	0.337*	L_PH_th	−0.359*	L_lOFC_th	−0.350*		
R_iCC_th	0.401*	R_ThaMD	−0.404**	L_raCC_th	−0.338*		
R_ThaMD	−0.375*	L_HippMD	−0.474**	R_iCC_th	−0.328*		
L_HippMD	−0.466**	R_HippMD	−0.493**	Perseverative Errors			
R_HippMD	−0.418**	2 s		L_Hipp_vol	−0.346*		
V Form		For_FA	0.451**	R_Hipp_vol	−0.313*		
For_FA	0.440**	L_Cing_FA	0.535**	L_lOFC_th	−0.335*		
R_Cing_FA	0.296*	L_Cing_MD	−0.288*	NonPerseverative Errors			
R_Cing_MD	−0.390**	R_Cing_FA	0.452**	L_lOFC_th	−0.319*		
R_UF_vol	0.349*	R_Cing_MD	−0.293*	R_HippMD	0.327*		
R_UF_MD	−0.312*	L_UF_FA	0.493**	Failure to Maintain			
R_Tha_vol	0.432**	L_UF_MD	−0.289*	R_pCC_th	0.350*		
L_lOFC_th	0.341*	R_UF_FA	0.344*	L_PH_th	0.326*		
L_raCC_th	0.379*	R_UF_MD	−0.319*	L_EC_th	0.369*		
R_caCC_th	0.352*	L_PH_th	−0.415**	R_EC_th	0.368*		
R_iCC_th	0.357*	R_ThaMD	−0.361*	R_ThaMD	−0.344*		
L_HippMD	−0.447**	L_HippMD	−0.385*				
R_HippMD	−0.424**	R_HippMD	−0.460**				

*Indicates p < 0.05 and **p < 0.01.

*adj, adjusted for intracranial volume; Amy, Amygdala; ca, caudalanterior; CC, cingulate cortex; Cing, cingulum; DI, discrimination index; EC, entorhinal cortex; FA, fractional anisotropy; For, Fornix; Hipp, hippocampus; i, isthmus; LDCR, long-delayed cue recall; LDFR, long delayed free recall; L, left hemisphere; l, lateral; m, medial; MD, mean diffusivity; OFC, orbitofrontal cortex; p, posterior; PH, parahippocampal; R, right hemisphere; ra, rostralanterior; SDCR, short-delayed cue recall; SDFR, short-delayed free recall; Tha, thalamus; UF, uncinate fasciculus; vol, volume*.

### California Verbal Learning Test II (CVLT II)

California Verbal Learning Test II evaluates episodic verbal learning and memory (24). In our study, the fornix, bilateral uncinated fasciculus (UF) bilateral thalami, left parahippocampus, and right posterior cingulate cortex were found to be critical regions for encoding function. For short term recall, our analyses showed significant correlations for right anterior and posterior cingulate cortices and thalamus whereas for long-term recall; bilateral thalami, and bilateral anterior and posterior cingulate cortices. Fornix was found to be crucial for both short and long term recall.

Recall discriminability measures, which we recently included in CVLT version II for better recall accuracy, were shown to be related with most of the limbic gray and white matter structures bilaterally; frontal limbic cortices, bilateral anterior and posterior cingulate cortices, bilateral thalami, fornix and right uncinate and cingulum tracts. Please see Table 3 for the numeric results and Figures 3A–E for the prominent scatter plots.

### The Brief Visuospatial Memory Test—Revised

Brief Visuospatial Memory Test—Revised has been commonly used to evaluate visuospatial memory abilities in neuropsychological populations (25). In our MS population, visuospatial recall is found to be related to fornix, right UF, right anterior, and posterior cingulate cortices whereas learning to right cingulum, left UF, left EC. Delayed recall scores showed correlations with right UF, right anterior, and posterior cingulate cortices.

Although verbal recall discrimination is found to be carried out by more structures in LS, visuospatial recall discrimination is found to be related to bilateral anterior and posterior cingulate cortices bilaterally and right PH cortex. Table 3 and Figures 4E,F illustrates numeric results and notable scatter plots, respectively.

### Controlled Oral Word Association Test

Controlled oral word association test evaluates phonemic fluency with FAS and semantic fluency with animal’s test (27). Previously (41), phonemic and semantic fluency are related to temporal regions. In our study, main limbic pathways connecting frontal and temporal lobes are shown to correlate with verbal phonemic and semantic fluency as well as right anterior and posterior cingulate cortices. Interestingly, bilateral thalami showed more association to semantic VF than phonemic VF (Table 3; Figure 3F).

### Symbol Digit Modality Test

Symbol Digit Modality Test is a useful screening test for CI in MS and assesses attention, visual scanning, and motor speed (22), in our cohort; fornix, right cingulum, left OFC, and right posterior cingulate cortex, we found to be associated with the SDMT score (Table 3; Figures 4A,B).

### Paced Auditory Serial Addition Test

Paced Auditory Serial Addition Test measures auditory information processing speed and flexibility, as well as calculation ability (21). All the main limbic white matter pathways, bilateral hippocampi, left PH cortex, and right thalamus were found to be associated with PASAT (Table 3; Figures 4C,D).

### Benton Judgment of Line Orientation (JLO)

Judgment of Line Orientation is a standardized test of visuospatial skills (27) commonly associated with functioning right parietal and occipital activation, as well as bilateral frontal activation (42). In our MS population, although it was found to be lateralized to right cingulum and UF, and to left OFC, overall it showed bilateral associations to gray and white matter limbic structures. Fornix, bilateral cingulate cortices, bilateral hippocampi, and thalami were the most prominent structures related to the scores (Table 3).

### Wisconsin Card Sorting Test

Wisconsin Card Sorting Test scores test attention, working memory, and visual processing. These scores measure frontal lobe functions; strategic planning, organized searching, utilizing environmental feedback to shift cognitive sets, directing behavior toward achieving a goal, and modulating impulsive responding (26). As expected, OFC showed correlation but lateralized to left. Temporal lobe structures were also found to be involved; bilateral hippocampi and entorrhinal cortices as well as left PH cortex. Bilateral cinguli and fornix as well as right thalamus showed significant correlations with the functions tested (Table 3).

### Fornix

Our data indicate forniceal white matter to be essential for multimodal cognitive functions. It might be due to its central location connecting important limbic centers.

### Cinguli/Cingulate Cortices (CC)

Left cingulum was found to be associated with visual spatial memory, phonemic fluency, executive functions, and processing speed whereas right cingulum similar to fornix was associated with broader cognitive functions such as executive functions, attention motor and processing speed, phonemic and semantic fluency, visuospatial learning and skills, and verbal learning. When it comes to parts of cingulate cortices, the isthmus of right CC showed correlation with all cognitive modalities; right anterior caudal CC with visuospatial memory and skills, verbal learning, semantic fluency; isthmus of left CC with visuospatial skills and verbal memory, and left anterior caudal CC with executive functions, visuospatial skills, and verbal learning. Posterior CC was associated with verbal learning bilaterally and executive functions on the right side.

### Uncinate Fasciculus

Left UF showed significant correlation with verbal and visuospatial learning, phonemic fluency, and processing speed whereas right UF with visuospatial memory and skills, verbal learning, phonemic and semantic fluency, and processing speed.

### Orbitofrontal Cortex

Left lateral OFC correlated with written and oral attention, executive functions, whereas right lateral OFC with verbal learning and right medial OFC with visuospatial learning and memory.

### Hippocampus/Parahippocampus

Right hippocampus was microstructurally related to visuospatial skills, executive functions and processing speed, left hippocampus related to phonemic and semantic fluency, executive functions and processing speed. Bilateral parahipppocampi related to visual and verbal learning, and left parahippocampus with processing speed and executive functions.

### Thalamus

Left thalamus was found to be correlated with verbal learning, visuospatial skills, semantic fluency whereas right thalamus with verbal learning, visuospatial skills, processing speed and executive functions, phonemic and semantic fluency.

### Amygdala

Bilateral amygdala were associated with verbal recognition discrimination.

### Entorhinal Cortex

Bilateral entorhinal cortices were correlated with executive functions and left entorhinal with verbal recognition discrimination.

## Discussion

This study provided associations between LS structures including volumes and microstructure measures with various cognitive functions, both evaluated in a comprehensive manner for the purpose of a detailed cognitive mapping of limbic regions in MS patients with and without CI. Our findings are mostly in line with previous less comprehensive MS studies as can be seen in Table 1. The novelty of this study is the adoption of a comprehensive approach to the role of all major limbic structures in different cognitive functions rather than studying one or some limbic structures and their cognitive functions. Results show that many limbic gray and white matter structures are involved in various cognitive functions rather than one specific center being responsible of a specific function. We adjusted our correlations for age, LL and education, which play independent roles in cognitive functioning of patients. Overall, fornix and cingulum/cingulate cortices were found to be the strongest correlates of CI in MS.

Highlights of our findings are that cingulate cortex and its main white matter pathway the cingulum, which have multiple complex functions that make its exact behavioral significance very elusive but are known to mainly serve as a modulator among different cognitive networks (7). In our cohort, similar to the literature, the cingulate cortex especially its anterior part, showed correlation with multi cognitive modalities evaluated by the MACFIMS. Interestingly, although posterior cingulate is known to be involved with visuospatial awareness and memory, our MS cohort showed association to verbal learning and executive functions. Like fornix, cingulum was associated with broader cognitive functions. A previous study revealed that cingulum DTI metrics were the best classifiers across numerous cognitive tests measuring CI in MS [(43); see also Table 1].

Although classically hippocampal formation is responsible for semantic memory formation and spatial mapping, it also plays a critical role in modulating attention, reactivity to external cues, behavioral flexibility. Previously, gray matter loss in hippocampus was shown to be related with overall CI in MS (44, 45). Our analyses showed that injury to hippocampal formation led to more cognitive slowing more than verbal and visual learning. Also other studies have shown hippocampal associations with verbal memory on the left (46, 47), and spatial memory on the right in MS (47). We have not observed any lateralization in our cohort except for verbal recognition discrimination lateralized to the left EC also shown in a previous study (48). Our findings for lateralization should be interpreted cautiously as we had several left handed (3) subjects in our cohort.

In contrast to limited functions of hippocampus in our MS cohort, its main output the fornix, was a predictor of multimodal CI. It is not surprising that injury to fornix leads to such widespread effect on cognition as it is one of the more prominent pathways in the CNS and serves as bridge between most limbic structures (7). Previous MS studies support our findings showing fornix to be related to various cognitive functions (4) such as visuospatial memory (10, 49), verbal memory (11, 49), and delayed recognition (50).

Thalamus showed widespread associations with multimodal CI in our cohort similar to previous studies (13, 14). Some other studies in MS patients showed association between thalamus and attention/executive functions (12, 14, 45) verbal memory (12).

Lesions affecting amygdala (i.e., Kluver–Bucy syndrome) are known to cause loss of insight and inability of recognizing one’s self socially inappropriate behaviors (51). In parallel, in our study, degeneration in bilateral amygdala were associated with failure verbal recognition discrimination. OFC is classically known as top–down execution modulator of the limbic networks (8). It was previously shown to be related with overall CI in MS (48). In our cohort, language-related attention and executive functions mainly lateralized to left whereas visuospatial learning and memory lateralized to right. This can be explained by the fact that the right hemisphere is more involved with visuospatial awareness, whereas the left with language related functions. As the main white matter pathway connecting OFC to LS, the correlation profile of uncinate fasciculus and CI was similar to OFC without lateralization though. Our findings are somewhat in parallel with previous studies showing the role of UF in visuospatial memory (10), processing speed (11) in MS patients.

## Conclusion

A more detailed cognitive mapping of the LS is important in understanding functional neuroanatomy of common cognitive domains affected by MS. It also provides a non-invasive, *in vivo* marker for possibly assessing cognitive recovery and designing more targeted treatment approaches for CI. To the best of our knowledge, our study is the first to have comprehensive cognitive mapping of the LS in an MS cohort, previous studies have limited correlations between specific structures and cognitive domains. Our cross-sectional study is limited by a relatively small sample size, lack of healthy subjects, and heterogeneity of the MS groups. Also most of the significant correlations in our study did not survive multiple comparisons possibly due to small sample size; however, false positivity cannot be ruled out. Longitudinal prospective studies with MS patients enrolled soon after diagnosis and monitored by MRI and cognitive testing for progression would more clearly reveal the role of LS in the MS related CI. I Larger clinical studies with adequate sample size will provide further exploration in the role of LS and other brain structures in MS, such studies are in the planning phase.

## Ethics Statement

Written informed consent was obtained from each subject following University of Texas Health Science Center Institutional Review Board approval of the protocol.

## Author Contributions

ZK—writing and preparation of the manuscript and MRI and statistical analyses; KH and BM—MRI and statistical analyses and critical revision of the manuscript for important intellectual content; KY, MK-K, AK, JL, FN—critical revision of the manuscript for important intellectual content.

## Conflict of Interest Statement

The authors declare that the research was conducted in the absence of any commercial or financial relationships that could be construed as a potential conflict of interest.
